# Reverse boot technique for applying traction in below knee amputees

**DOI:** 10.1308/rcsann.2024.0046

**Published:** 2024-06-14

**Authors:** R McAllister, M Franklin, N Hyder

**Affiliations:** Leighton Hospital, Crewe, UK

## Background

Traction is often required for adequate reduction of neck of femur fractures requiring fixation. One method to achieve this is placing the ankle securely into a boot that is part of a traction table.^1^ This is not possible when the patient has had a below knee amputation (BKA).

We describe a technique to apply traction to the residual limb in patients with BKAs.

## Technique

**Figure 1:**
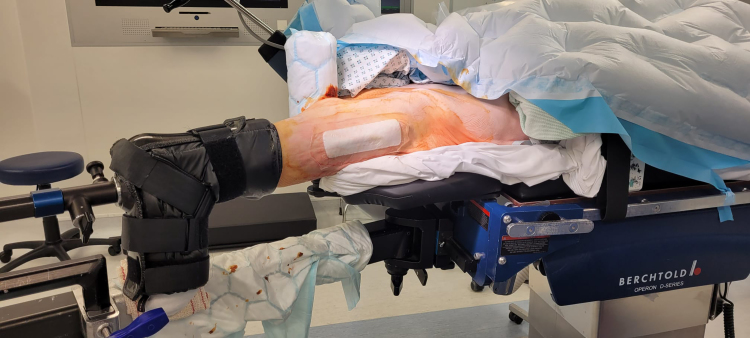
Clinical photograph demonstrating the reverse boot technique

The patient is transferred onto the traction table. The contralateral leg and torso are set up in the usual manner. The ipsilateral knee and proximal tibial stump are protected with synthetic wool. The boot is rotated 180 degrees and the patient's knee is placed into the boot with the knee at 90 degrees of flexion and sitting where a heel would normally sit in a boot. The straps of the boot are fastened and traction can be applied (Figure 1).

## Discussion

Traction on the leg assists with reduction of the limb undergoing fracture fixation. Methods of applying traction to a BKA stump include using a Steinmann pin in the proximal tibia or distal femur.^2^ This is effective for applying traction but may be associated with morbidity such as infection and fracture.

This method provided support of the limb and allowed for the application of traction to reduce the fracture while the dynamic hip screw was inserted without the morbidity associated with other techniques. Although this technique has been described previously^3–5^ we believe that it is not widely recognised and should be considered when faced with this scenario.
